# Ablation Parameters Predicting Pulmonary Vein Reconnection after Very High-Power Short-Duration Pulmonary Vein Isolation

**DOI:** 10.3390/jcdd11080230

**Published:** 2024-07-24

**Authors:** Márton Boga, Gábor Orbán, Zoltán Salló, Klaudia Vivien Nagy, István Osztheimer, Arnold Béla Ferencz, Ferenc Komlósi, Patrik Tóth, Edit Tanai, Péter Perge, Béla Merkely, László Gellér, Nándor Szegedi

**Affiliations:** Heart and Vascular Centre, Semmelweis University, Városmajor u. 68., 1122 Budapest, Hungary

**Keywords:** pulmonary vein isolation, pulmonary vein reconnection, very high-power short-duration, interlesion distance, generator impedance

## Abstract

Background: Recurrences due to discontinuity in ablation lines are substantial after pulmonary vein isolation (PVI) with radiofrequency ablation for atrial fibrillation. Data are scarce regarding the durability predictors for very high-power short-duration (vHPSD, 90 W/4 s) ablation. Methods: A total of 20 patients were enrolled, who underwent 90 W PVI and a mandatory remapping procedure at 3 months. First-pass isolation (FPI) gaps, and acute pulmonary vein reconnection (PVR) sites were identified at the index procedure; and chronic PVR sites were identified at the repeated procedure. We analyzed parameters of ablation points (n = 1357), and evaluated their roles in predicting a composite endpoint of FPI gaps, acute and chronic PVR. Results: In total, 45 initial ablation points corresponding to gaps in the ablation lines were analyzed. Parameters associated with gaps were interlesion distance (ILD), baseline generator impedance, mean current, total charge, and loss of catheter–tissue contact. The optimal ILD cut-off for predicting gaps was 3.5 mm anteriorly, and 4 mm posteriorly. Conclusions: Biophysical characteristics dependent on generator impedance could affect the efficacy of vHPSD PVI. The use of smaller ILDs is required for effective and durable PVI with vHPSD compared to the consensus targets with lower power ablation, and lower ILDs for anterior applications seem necessary compared to posterior points.

## 1. Introduction

Pulmonary vein isolation (PVI) with radiofrequency (RF) ablation is established as an effective treatment for atrial fibrillation (AF); still, AF recurrence is substantial after ablation procedures. Pulmonary vein reconnection (PVR) underlies the majority of these recurrences, evidenced by acute PVR at initial procedures and chronic PVR found at repeated procedures, which occurs due to either insufficient lesion volumes or discontinuity in the ablation line [1]. Consequently, the key determinants of durable PVI and freedom from recurrence hinge on accurately predicting lesion volumes and ensuring continuous ablation lines. Lesion volumes can be estimated by lesion prediction indices such as the ablation index (AI), and continuity can be achieved by the adequately close placement of ablation points, marked by interlesion distance (ILD). The optimal AI and ILD targets have been clearly established for low-power ablation [2,3,4]. However, AI is not available for very high-power short-duration (vHPSD, 90 W/4 s) ablation and data are scarce on parameters influencing the acute and chronic efficacy of 90 W PVI. Recent studies have demonstrated that vHPSD results in smaller lesions (in both width and depth) than lower power ablation [5]. This highlights the necessity to reevaluate the ILD targets for 90 W ablation. Although baseline generator impedance and RF current have also been suggested to impact the lesion volumes of RF ablation (by lower circuit impedance resulting in higher current and larger lesion dimensions) [6,7,8], their role in the contiguity of ablation lines has never been evaluated before.

We hypothesized that vHPSD requires smaller ILDs compared to lower power settings to achieve effective and durable PVI; furthermore, higher baseline generator impedance and lower mean current of ablation points may result in a higher probability of PVR. Thus, we aimed to analyze parameters of ablation points including ILD, impedance and current of vHPSD procedures, and evaluate their roles in predicting first-pass isolation (FPI) gaps, acute PVR at initial procedures, and chronic PVR assessed by repeated electrophysiological evaluation.

## 2. Materials and Methods

### 2.1. Patient Population

Patients undergoing 90 W PVI were enrolled in the study. Three months following the initial procedure, left atrial high-density mapping was performed in all subjects to assess the long-term durability of PVI, as a part of the “HPSD Remap” study (URL: ClinicalTrials.gov; Unique identifier: NCT05459831) [9]. All participants provided written informed consent to the ablation and remap procedures, data retrieval and analysis. Ethics approval was obtained from the Hungarian National Public Health and Medical Officer Service (9119-2/2022/EÜIG).

### 2.2. Initial Ablation Procedure

**Figure 1:**
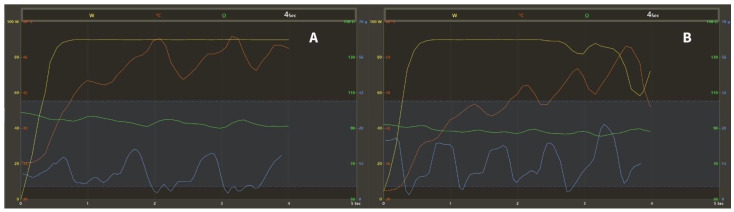
Graph showing the changes of ablation parameters during the 4-s RF applications with the QMODE+ setting (90 W/4 s, temperature-controlled mode). Yellow: power (W); red: temperature (°C); blue: contact-force (g), dashed lines: target contact-force range (5–40 g); green: impedance (Ω). (**A**) The target temperature of 55 °C is not reached, stable 90 W of power is delivered for 4 s. (**B**) The target temperature is reached, power is downregulated to prevent overheating. RF = radiofrequency.

Before the procedure, all patients underwent either contrast-enhanced left atrial computed tomography or transesophageal echocardiography to rule out left atrial appendage thrombus. Procedures were conducted under conscious sedation and performed by experienced operators. Patients were receiving non-Vitamin K antagonist oral anticoagulants, of which a single dose was omitted on the morning of the procedure. Mild (conscious) sedation with midazolam, propofol and fentanyl was utilized in all procedures. Local anesthesia was used for femoral venous access. A double transseptal puncture was performed guided by fluoroscopy and pressure monitoring. Following the transseptal puncture, intravenous unfractionated heparin boluses were administered to maintain an activated clotting time of more than 300 s. A fast anatomical map of the LA was obtained with the CARTO mapping system (CARTO 3, Biosense Webster Inc., Diamond Bar, CA, USA), along with a multipolar mapping catheter (either Lasso or PentaRay, Biosense Webster Inc., Diamond Bar, CA, USA). Subsequently, point-by-point radiofrequency (RF) applications were delivered around the ipsilateral PVs with the QDOT Micro catheter (Biosense Webster Inc., Diamond Bar, CA, USA) in the QMODE+ setting (90 W/4 s, temperature-controlled mode; Figure 1). The QDOT Micro catheter is equipped with contact-force sensing and six thermocouples embedded in the tip to ensure precise local temperature measurement. The ablation catheter was used along with a steerable sheath (either Agilis, Abbott, Chicago, IL, USA, or Vizigo, Biosense Webster Inc., Diamond Bar, CA, USA).

**Figure 2:**
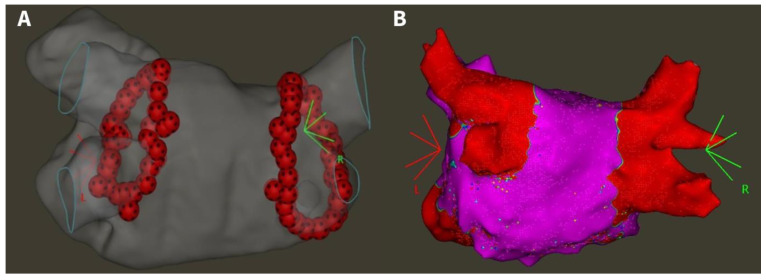
(**A**) Electroanatomical map of 90 W pulmonary vein isolation (posteroanterior view). (**B**) High-density voltage map of a patient with four isolated pulmonary veins at the repeat procedure (posteroanterior view) [9]. Red area on right panel: bipolar voltage < 0.5 mV; purple area: bipolar voltage > 0.5 mV.

During PV encirclement, the mapping catheter was positioned in the contralateral PVs to blind the operator to the presence or absence of PV potentials. Following the completion of the ablation circle, first-pass isolation (FPI) was evaluated with the multipolar mapping catheter. If absence of entrance block was evident, touch-up applications were delivered with the same power setting to reach complete isolation of all PVs. After a 20-min waiting period, acute PVR was assessed for all PVs. In case of acute reconnection, additional applications were delivered to reisolate the veins. Ablation points at the gap locations in the first-pass circle and acute PV reconnection sites were annotated. No additional ablation was performed beyond PVI. A representative electroanatomical LA map after 90 W PVI is shown in Figure 2A.

### 2.3. Repeat Electrophysiology Study

All patients underwent electrophysiology mapping 3 months after the initial procedure, irrespective of clinical symptoms. Left atrial access was obtained with the same technique used at the initial ablation. High-density voltage and activation maps were created during distal coronary sinus pacing using the CARTO system and a multipolar catheter (PentaRay or OctaRay). The PVs were evaluated for PVR based on the high-density map, comprising a minimum of 2000 points evenly distributed across all LA sites (Figure 2B). The exact locations of PVR were marked and carefully compared to the maps of initial procedures to identify the initial ablation points at reconnection sites.

### 2.4. Data Collection

The parameters of all ablation points of initial procedures were collected for analysis from the CARTO system. These parameters were: application time (t_appl_, s); mean power (P_mean_, W); maximum temperature (T_max_, °C); baseline generator impedance (Z_gen_, Ω); impedance drop (ID, Ω); minimum CF (CF_min_, g); mean CF (CF_mean_, g); maximum CF (CF_max_, g) and inter-lesion distance (ILD, mm) of neighboring points. We calculated the following parameters for ablation points: total RF energy delivered (E_total_, J); mean RF current applied (I_mean_, A); and total charge delivered (Q_total_, C) according to the given formulas.

$$E_{total}=P_{mean}\times t_{appl}$$

$$I_{mean}=\sqrt{\frac{P_{mean}}{Z_{gen}}}$$

$$Q_{total}=I_{mean}\times t_{appl}$$

Marked ablation points at the gap locations in the first-pass circle, acute and chronic PVR sites were identified, and touch-up applications at these locations were omitted during data collection. The anterior/posterior and right/left sided location of ablation points were also registered. Intermittent loss of contact (LOC) was evaluated for all ablation points, defined by a minimum contact force of 0 g.

### 2.5. Statistical Analysis

Continuous variables are reported as either mean and standard deviation or median and interquartile range. The distribution of variables was tested with the Shapiro–Wilk test. For the comparison of unpaired groups, the Mann–Whitney U test was used, as all variables showed non-parametric distribution. Categorical variables are reported as frequency and percentage and were compared by Fisher’s exact test. Optimal cut-off values were established based on receiver operating characteristic (ROC) analysis, odds ratios (OR) and Fischer’s exact *p*-values. Univariate logistic regression was performed to identify predictive variables for gaps. Variables with the greatest independent effect were identified using multivariable logistic regression analysis. Statistical analyses were conducted using GraphPad Prism 10 (GraphPad Softwares Inc., San Diego, CA, USA). A significance level of *p* < 0.05 (two-tailed) was considered statistically significant.

## 3. Results

### 3.1. Study Population and Initial Procedural Characteristics

A total of 20 patients were enrolled in the study, undergoing 90 W PVI and repeated electrophysiological study 3 months after the index procedure. The baseline parameters of the study population are presented in Table 1. Mean age was 65 ± 8 years, 45% were female, and 50% of patients had persistent AF. Mean procedure time was 75.6 ± 12.9 min and bilateral FPI was achieved in 80%. No major complications occurred.

### 3.2. Incidence and Location of Gaps and Reconnections

**Figure 3:**
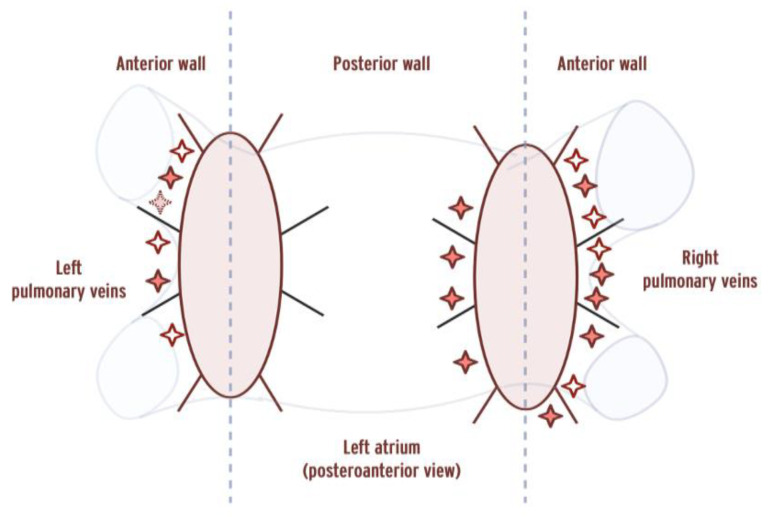
Location of gaps on a 16-segment pulmonary vein ostium model. Empty stars indicate gaps in the first-pass circle, dashed stars indicate acute PVR, and bold stars indicate chronic PVR. PVR = pulmonary vein reconnection. Vertical dashed lines indicate the border between anterior/posterior segments.

A total of 1357 ablation points were analyzed. FPI gaps were present in 4 patients at a total of 7 sites corresponding to 19 ablation points, acute PVR occurred in 1 patient at 1 site corresponding to 6 ablation points, while chronic PVR was present in 4 patients at a total of 11 sites corresponding to 20 ablation points. In total, six patients had gaps of any kind (acute, chronic PVR or FPI gaps). The location of gaps around the PV ostia are presented in Figure 3. Ablation points corresponding to gaps were located more frequently at anterior segments (OR = 2.526, *p* = 0.0083), while right-sided location showed a trend towards a higher probability of gaps (OR = 1.781, *p* = 0.0722).

### 3.3. Ablation Point Parameters

**Figure 4:**
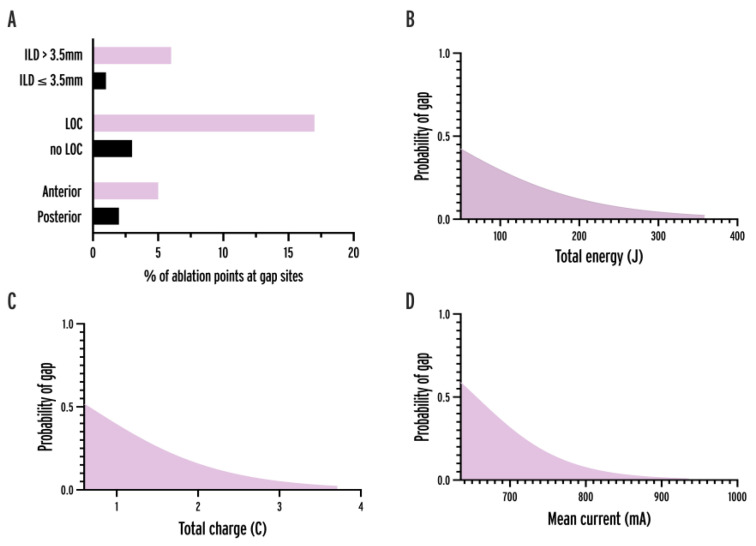
Association between variables and gap probability. (**A**) Bar chart showing the percentage of ablation points at gap sites in the case of anterior location, loss-of-contact, and ILD > 3.5 mm. (**B**–**D**) Logistic regression curves of total energy, total charge, and mean current delivered by ablation point. ILD = interlesion distance, LOC = loss-of-contact.

The parameters of ablation points at isolated sites were compared to ablation points at gap sites (Table 2, Figure 4). In comparative analysis, the parameters showing significant differences at isolated vs. gap sites were ILD (3.3 vs. 4 mm, *p* < 0.0001), baseline generator impedance (112 vs. 114 Ω, *p* = 0.0179), mean current (858.4 vs. 854.7 mA, *p* = 0.0055), total charge (3.43 vs. 3.4 C, *p* = 0.0044), and LOC (2.7 vs. 13.3%, *p* = 0.0020). In univariate logistic regression analysis, significant predictor variables were ILD, application time, mean power, baseline generator impedance, total energy, mean current, total charge, LOC and anterior location.

### 3.4. Optimal ILD Cut-Offs

**Figure 5:**
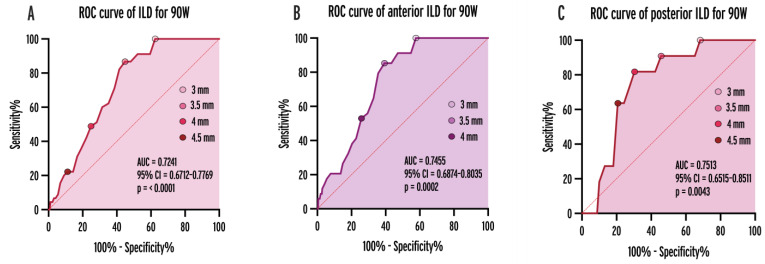
ROC curves of ILDs for predicting gaps and reconnections in the case all applications (**A**), anterior (**B**), and posterior (**C**) ablation points. AUC = area under curve, CI = confidence interval, ILD = inter-lesion distance, ROC = receiver operating characteristic.

To determine the optimal cut-off value of ILDs for predicting gaps, we performed receiver operating characteristic (ROC) analysis (Figure 5, AUC = 0.6052, 95% CI = 0.5217–0.6886, *p* = 0.0163). An overall ILD of 3.5 mm was an optimal cut-off. The probability of gaps and reconnections was significantly higher in case of ILDs > 3.5 mm (OR = 6.238; 95% CI = 2.939–13.24, *p* < 0.0001). When analyzing the cut-offs for anterior and posterior applications separately, the optimal cut-point was 3.5 mm (OR = 6.614, *p* < 0.0001) and 4 mm (OR = 8.711, *p* = 0.0007), respectively.

### 3.5. Atrial Rhythm during Ablation

A total of nine patients (45.5%) were in AF during ablation. Comparing ablation points created during ongoing AF (n = 610) to ablation points during sinus rhythm (n = 747), the measured maximum temperature, mean CF, applied current were higher (48.3 vs. 46 °C, *p* < 0.0001; 17 vs. 14 g, *p* < 0.0001; and 864.7 vs. 855.7 mA, *p* < 0.0001; respectively), and the incidence of LOC was lower (0.3% vs. 3.8%, OR = 0.3783, *p* = 0.0008). Meanwhile, the incidence of gaps associated with the given ablation points was similar between applications during AF and sinus rhythm (3.74% vs. 3.2%, *p* = 0.7125).

### 3.6. Multivariable Analysis

Variables showing significance (*p* < 0.05) were incorporated into multivariable logistic regression models to identify predictors with the greatest independent effect. As t_appl_, P_mean_ and Z_gen_ were used to calculate the values of E_total_, I_mean_ and Q_total_, these variables showed highly significant multicollinearity (R^2^ > 0.99, VIF > 200, *p* < 0.0001). Therefore, 6 separate models were run, each including only one of the above variables besides ILD > 3.5 mm, LOC and anterior location of ablation points (Table 3). All of the variables remained independently predictive in the models, but out of the six highly correlated variables, Q_total_ had the lowest *p*-value (0.0002) and the best predictive power (AUC = 0.7452) of the given models. LOC had the greatest effect on the likelihood of gaps out of all predictors.

## 4. Discussion

### 4.1. Main Findings

Our results show that the determinants of the contiguity of ablation lines created during vHPSD PVI are ILD, stable catheter–tissue contact, delivered energy, and also current and charge which are influenced by the generator impedance. The maximum temperature measured by the QDOT catheter was not associated with gaps and reconnections. The use of smaller ILDs is required for effective and durable PVI with vHPSD compared to consensus targets for lower power settings. As the probability of gaps at the anterior wall is greater with the fixed application time of 90 W/4 s ablation, the use of lower ILDs for anterior applications seems necessary compared to posterior points.

### 4.2. Lesion Formation with High RF Power Settings

Compared to LPLD ablation, higher RF current is delivered within a shorter timeframe during application of higher power. This approach aims to induce more resistive and minimal conductive heating, leading to more consistent lesion formation. Initial animal studies suggested that lesions generated with vHPSD are wider with similar depth [10,11]; however, subsequent studies challenged this result [5]. Nakagawa et al. demonstrated that lesions created using higher power settings have smaller maximum width and maximum depth (7.9/3.6 mm with 90 W/4 s vs. 8.2/4.8 mm with 50 W/10 s vs. 8.7/5.6 mm with 30 W/30 s), with 90 W lesions being the smallest in volume [5].

### 4.3. Optimal Inter-Lesion Distance for vHPSD Ablation

Recent studies indicating that higher power results in narrower and shallower lesions underscore the need for a reevaluation of ILD targets during vHPSD PVI. In one animal study, all lines created around the right superior PV of swine in vHPSD mode with ILDs of 3–4 mm were blocked at 1-month electrophysiological evaluation [11]. Another investigation compared 3, 4, 5 and 6 mm ILDs for 90 W ablation of intercaval lines in adult sheep (with maximal tissue thickness comparable to human PV ostia) [12]. The study reported that with vHPSD, only 3 mm and 4 mm ILDs resulted in durable block at 21-days, while 5–6 mm ILDs did not.

The difference between ILD values in this article and in the original HPSD remap paper requires an explanation. In the original paper the maximal ILD target values were specified [9]; however, in the current paper we report the “actual” ILD values. The actual ILD is essentially lower than the target ILD, as in case of neighboring lesions with an ILD larger than the target, we need to deliver an additional application between those lesions.

In our study, the optimal cut-point of ILDs was 3.5 mm all-around. This is lower than the widely accepted 6 mm cut-off of the CLOSE protocol used for low-power ablation [3]. This suggests that the use of smaller ILDs is required for effective and durable PVI with vHPSD compared to lower power ablation, as a result of smaller lesion volumes. As the application time is set at a fixed value of 4 s with 90 W ablation, the use of lower ILDs for anterior applications seems necessary compared to posterior points, with an optimal cut-point of 3.5 mm anteriorly and 4 mm posteriorly. These somewhat low ILD targets similar to those used in the FAST AND FURIOUS PVI study [13], might be explained by the need for a greater overlap for smaller lesions to provide effective coverage of deep epicardial regions in the left atrial wall. Furthermore, the lesion depth with vHPSD may be larger in case of consecutive points with greater overlap, which could help in creating transmural lesions [14].

### 4.4. Impedance, Applied Current and Charge

The rationale behind using power as a measure of heat-inducing capability of RF ablation is grounded in Joule’s law of thermodynamics; according to which, the thermal energy produced within the RF circuit can be calculated using the formula:

$$E_{thermal}={I_{mean}}^{2}\times Z_{gen}\times t_{appl}=E_{total}=P_{mean}\times t_{appl}$$

However, it is crucial to recognize that generator impedance encompasses not only local myocardial impedance, which influences effective resistive heating and lesion formation, but also includes all tissues between the catheter tip and neutral electrode. Therefore, it is important to know how much power is absorbed around the catheter and how much in the rest of the tissues before reaching the neutral electrode. In an in silico study on RF power delivery in 3-dimensional full thorax models, around 75% of the power was absorbed within a 2 cm-radius sphere around the catheter tip, and 25% by the rest of the tissues [15]. Therefore, epicardial, mediastinal and subcutaneous fat which varies greatly between patients, can in theory significantly reduce the ratio of power absorbed in the myocardium, due to the high impedance of adipose tissue. Likewise, the positioning of the neutral electrode can also exert a meaningful impact on generator impedance [16]. Consequently, the association between applied power, total energy and lesion volumes is influenced by many factors altering circuit impedance like bodyfat percentage, and neutral electrode positioning [8], which should be important factors to consider during RF ablation procedures. Based on these considerations, applied current might be a more accurate measure of lesion formation than power or total energy. The square of the current applied to the myocardium is inversely proportional to circuit impedance, which can result in variability of lesion volumes from patient to patient, but also between ablation points of the same procedure. Total charge delivered, which is calculated by multiplying current and application time is also proportional to the total generated heat, and is similarly influenced by generator impedance and its determinant factors.

In the current study, higher baseline generator impedance was a significant predictor for gaps, while mean current and total charge showed an even higher significance. These factors are not displayed by the CARTO system, but they could be useful parameters for lesion prediction in the absence of AI with vHPSD. Also, changing temperature guided power modulation to current modulation could have the potential to further enhance the efficacy of 90 W ablation [8,17]. This could also be a step towards enhancing the cerebral safety of vHPSD ablation, which raised concerns previously [18].

### 4.5. Catheter–Tissue Contact, Stability and Contact-Force

Catheter–tissue contact, active electrode coverage and stability can be characterized by CF values. These factors influence current density and the distribution of the total produced heat. In the case of catheter sliding and LOC, the produced heat is not concentrated well within an ablation point, that could lead to insufficient lesion volumes or inappropriate lesion geometry. Higher CF values indicate more electrode surface coverage and result in more current delivered to the myocardium [19,20]. We have shown that transient LOC is much more strongly associated with insufficient lesion formation than overall CF. Catheter–tissue contact is a crucially important factor in the case of 90 W/4 s ablation, where even a momentary LOC can result in a significant reduction in the current delivered to the myocardium. Our results also suggested that ablation during sinus rhythm results in a higher incidence of LOC, probably due to the atrial wall pushing the catheter tip during every beat.

### 4.6. Maximum Catheter Tip Temperature

Accurate maximal catheter tip temperature measurement is available with the QDOT catheter, which correlates with tissue temperatures, as the thermocouples are placed close to the catheter–tissue interface. Still, real catheter–tissue interface temperatures have been shown to be around 15 °C higher and tissue temperature at 3 mm depth 35 °C higher than the temperatures measured by the QDOT catheter [5]. Nakagawa et al. have reported that a more substantial portion of tissue temperature rise occurs following the termination of RF delivery (called “thermal latency”) with higher power compared to lower power settings, which is logical considering that conductive heating takes longer time than the short 4 s applications. Irrigation, blood flow and the pulsatility of atrial rhythm (sinus/AF) can also influence the measured temperature. In our study, the maximal temperature measured by the QDOT catheter only showed a trend for predicting gaps. For these reasons, the measured maximal temperature might not be the best parameter (in terms of efficacy) for guiding power delivery during vHPSD ablation.

### 4.7. Location of Gaps and Reconnections

Gaps and reconnections were more frequently located at anterior PV segments. While the mean lesion depth is around 3.6 mm with 90 W/4 s ablation [5], the minimum–maximum left atrial wall thickness measured on CT scans before PVI is 0.3–4.5 mm at anterior segments and 0.3–2.3 mm at the posterior wall [21]. Therefore, 90 W lesions might be too shallow at some parts of the anterior wall. Our results confirm this, as 75% of ablation points at gap sites were located anteriorly. This could potentially be overcome by reducing the ILDs at anterior segments and using the thermal latency, in the manner described above.

### 4.8. Limitations

This was a single-center study with four operating physicians, which might reduce the generalizability of the findings. Only 20 patients were enrolled in the study, as invasive electrophysiological remapping of asymptomatic patients is only reasonable in a low number of patients, from which sufficient data can be collected to reach statistical power. Matching the ablation points from the initial procedure to the gap locations on the voltage map created three months later might not be possible with 100% accuracy. This may limit the precision of the results.

## 5. Conclusions

The determinants of the contiguity of ablation lines created during vHPSD PVI included interlesion distance, stable catheter–tissue contact, delivered energy, and also current and charge which are influenced by the generator impedance. The use of smaller ILDs is required for effective and durable PVI with vHPSD compared to the consensus targets with lower power ablation. As the application time is set at a fixed value with vHPSD, the use of lower ILDs for anterior applications seems necessary compared to posterior points.

## Figures and Tables

**Table 1:** Baseline characteristics of the study population and initial procedural parameters.

Patient Characteristics (n = 20)	
Age, years	63 ± 7
Female, n	9 (45)
BMI, kg/m^2^	31.3 ± 2.1
Paroxysmal AF, n	10 (50)
Hypertension, n	11 (55)
Diabetes, n	5 (25)
Ischemic heart disease, n	1 (5)
LVEF, %	51.6 ± 5.6
LAVI, mL/m^2^	29.9 ± 9.4
Procedural Parameters (n = 20)	
Procedure time, min	75 ± 13
LA dwelling time, min	63 ± 10
Number of RF applications, n	85 ± 22
RF time, s	335 ± 85
Irrigation fluid, mL	215 ± 67
Bilateral FPI, n (%)	16 (80)

AF = atrial fibrillation, BMI = body-mass index, FPI = first-pass isolation, LA = left atrial, LAVI = left atrial volume index, LVEF = left ventricular ejection fraction, RF = radiofrequency.

**Table 2:** Parameters of ablation points at isolated sites and gap locations in the case of 90 W. Bold values indicate significant *p* (<0.05). CF = contact-force, CI = confidence interval, E_total_ = total energy applied, ID = impedance drop, ILD = inter-lesion distance, I_mean_ = mean current applied, LOC = loss-of-contact, OR = odds ratio, P_mean_ = mean power applied, Q_total_ = total current applied, t_appl_ = application time, T_max_ = maximum temperature, Z_gen_ = baseline generator impedance.

	Comparative Analysis			Univariate Logistic Regression Analysis		
	Points at Isolated Sites (n = 1311)	Points at Gap Sites (n = 45)	*p*-Value	OR	95% CI	*p*-Value
ILD, mm	3.3 (2.6–4.1)	4 (3.6–4.4)	**<0.0001**	2.139	1.576–2.939	**<0.0001**
t_appl_, s	4 (3.97–4)	3.9 (3.97–4)	0.0646	0.3936	0.2364–0.7156	**0.0006**
P_mean_, W	83 (83–84)	83 (83–84)	0.9591	0.9119	0.8547–0.9824	**0.0058**
E_total_, J	332 (330.3–334.2)	331.8 (329.6–332.7)	0.0629	0.9900	0.9844–0.9967	**0.0010**
I_mean_, mA	858.4 (842.3–871.4)	854.7 (834.4–864.1)	**0.0055**	0.9841	0.9755–0.9930	**0.0009**
Q_total_, C	3.43 (3.63–3.49)	3.4 (3.32–3.47)	**0.0044**	0.3238	0.1847–0.6058	**0.0001**
T_max_, °C	46.4 (44–49)	46 (43.7–48)	0.3407	0.9171	0.8370–1.001	0.0574
Z_gen_, Ω	112 (109–116)	114 (111–118.5)	**0.0179**	1.056	1.006–1.105	**0.0278**
ID, Ω	9 (7–11)	9 (7–11)	0.6714	0.9643	0.8624–1.044	0.4976
CF_min_, g	6 (3–10)	5 (3–8)	0.0930	0.9362	0.8683–1.001	0.0688
CF_mean_, g	14 (11–20)	14 (10.5–19)	0.5011	0.9849	0.9409–1.025	0.4868
CF_max_, g	25 (18–36)	25 (19–35)	0.8761	0.9917	0.9687–1.011	0.4460
CF_range_, g	18 (12–27)	19 (14.5–28.5)	0.4184	0.9981	0.9753–1.017	0.8579
LOC, n (%)	36 (2.7)	6 (13.3)	**0.0020**	5.453	1.976–12.87	**0.0003**
Anterior, n (%)	722 (55)	34 (75.6)	**0.0060**	2.526	1.310–5.264	**0.0083**
Right sided, n (%)	694 (52.9)	30 (66.7)	0.0936	1.781	0.9646–3.429	0.0722

**Table 3:** Multivariable logistic regression models for predicting gaps. *p*-values of the variables are presented for each model in which they are included. Bold values indicate the lowest P and AUC of individually selected variables (Q_total_). Indices show whether the variable has a positive (+) or negative (−) effect on the likelihood of gaps. The bottom line presents the AUC of each model. AUC = area under curve, E_total_ = total energy applied, ILD = inter-lesion distance, I_mean_ = mean current applied, LOC = loss-of-contact, P_mean_ = mean power applied, Q_total_ = total current applied, t_appl_ = application time, Z_gen_ = baseline generator impedance.

*p*-Value of Variables	Model 1 (t_appl_)	Model 2 (P_mean_)	Model 3 (E_total_)	Model 4 (I_mean_)	Model 5 (Q_total_)	Model 6 (Z_gen_)
ILD >3.5 mm	0.0049 ^+^	0.0062 ^+^	0.0052 ^+^	0.0065 ^+^	0.0056 ^+^	0.0038 ^+^
Anterior location	0.0010 ^+^	0.0011 ^+^	0.0010 ^+^	0.0024 ^+^	0.0013 ^+^	0.0017 ^+^
LOC	<0.0001 ^+^	<0.0001 ^+^	<0.0001 ^+^	<0.0001 ^+^	<0.0001 ^+^	<0.0001 ^+^
t_appl_	0.0005 ^−^					
P_mean_		0.0131 ^−^				
E_total_			0.0010 ^−^			
I_mean_				0.0017 ^−^		
Q_total_					0.0002 ^−^	
Z_gen_						0.04878 ^+^
**AUC of models**	0.7356	0.7206	0.7420	0.7435	**0.7452**	0.7238

+: The variable has a positive effect on the likelihood of gaps. −: The variable has a negative effect on the likelihood of gaps.

## Data Availability

The data will be shared upon request to the corresponding author.
